# A Low Selenium Level Is Associated with Lung and Laryngeal Cancers

**DOI:** 10.1371/journal.pone.0059051

**Published:** 2013-03-13

**Authors:** Katrzyna Jaworska, Satish Gupta, Katarzyna Durda, Magdalena Muszyńska, Grzegorz Sukiennicki, Ewa Jaworowska, Tomasz Grodzki, Mieczysław Sulikowski, Piotr Woloszczyk, Janusz Wójcik, Jakub Lubiński, Cezary Cybulski, Tadeusz Dębniak, Marcin Lener, Antoni W. Morawski, Karol Krzystolik, Steven A. Narod, Ping Sun, Jan Lubiński, Anna Jakubowska

**Affiliations:** 1 International Hereditary Cancer Centre, Department of Genetics and Pathology, Pomeranian Medical University, Szczecin, Poland; 2 Postgraduate School of Molecular Medicine, Warsaw Medical University, Warsaw, Poland; 3 Department of Otolaryngology and Laryngological Oncology, Pomeranian Medical University, Szczecin, Poland; 4 Department of General Thoracic Surgery, Pomeranian Medical University, Szczecin, Poland; 5 Maxillofacial Surgery, Pomeranian Medical University, Szczecin, Poland; 6 Department of Toxicology and Molecular Pathobiochemistry, Pomeranian Medical University, Szczecin, Poland; 7 Department of Water Technology and Environment Engineering, Institute of Chemical and Environment Engineering, West Pomeranian University of Technology, Szczecin, Poland; 8 Chair and Clinic of Ophthalmology, Pomeranian Medical University, Szczecin, Poland; 9 Women’s College Research Institute, Toronto, Ontario, Canada; The University of Texas M. D. Anderson Cancer Center, United States of America

## Abstract

**Purpose:**

It has been suggested that selenium deficiency is a risk factor for several cancer types. We conducted a case-control study in Szczecin, a region of northwestern Poland, on 95 cases of lung cancer, 113 cases of laryngeal cancer and corresponding healthy controls.

**Methods:**

We measured the serum level of selenium and established genotypes for four variants in four selenoprotein genes (*GPX1*, *GPX4*, *TXNRD2* and *SEP15*). Selenium levels in the cases were measured after diagnosis but before treatment. We calculated the odds of being diagnosed with lung or laryngeal cancer, conditional on selenium level and genotype.

**Results:**

Among lung cancer cases, the mean selenium level was 63.2 µg/l, compared to a mean level of 74.6 µg/l for their matched controls (p<0.0001). Among laryngeal cancer cases, the mean selenium level was 64.8 µg/l, compared to a mean level of 77.1 µg/l for their matched controls (p<0.0001). Compared to a serum selenium value below 60 µg/l, a selenium level above 80 µg/l was associated with an odds ratio of 0.10 (95% CI 0.03 to 0.34; p = 0.0002) for lung cancer and 0.23 (95% CI 0. 09 to 0.56; p = 0.001) for laryngeal cancer. In analysis of four selenoprotein genes we found a modest evidence of association of genetic variant in *GPX1* with the risk of lung and laryngeal cancers.

**Conclusion:**

A selenium level below 60 µg/l is associated with a high risk of both lung and laryngeal cancer.

## Introduction

Selenium is a trace element which is an essential component of several major metabolic pathways, including the antioxidant defense system and the immune system [1], [2]. Selenium intake varies, largely based on the selenium content of food. The mean selenium level of the population varies considerably between countries, with eastern European countries in general having levels that are much lower than those of Canada and the United States [1]. Several epidemiological studies have suggested inverse associations between serum selenium level and a number of different cancers, but the results have been inconsistent [3], [4]. The data for a protective effect against lung cancer are among the most compelling [3]–[10]. For many studies, the risk of cancer decreases with increasing serum selenium level, but the results of many other studies are negative and it is not clear if the dose-response curve is linear. In North America, where mean selenium levels are in the range of 120 ug/l - there may be less potential benefit to selenium supplementation than in countries where the mean level is low. The chemopreventive benefit of selenium may differ between individuals and the same dose may not be optimal for all. In supplementation trials, differences in the baseline level of selenium of patients or in the chemical form used as a selenium supplement (e.g. organic or inorganic) may also be important.

Some of the discrepancy in association studies might be explainable in part by the influence of genetic factors. Rayman suggests that genetic variants in selenoproteins may be relevant [1]. Selenium is incorporated as selenocysteine at the active site of a number of proteins (selenoproteins) [11], [12]. In humans there are 25 known selenoproteins, including four different glutathione peroxidases (*GPX1*, *GPX2*, *GPX3*, *GPX4*) and two thioredoxin reductases (*TXNRD1*, *TXNRD2*), but the function of many others is unknown [11], [12]. Selenoproteins are involved in different biological processes, ranging from DNA synthesis to protection against oxidative stress. The latter is assumed to be the probable link with cancer risk; i.e. cancer associated with selenium deficiency may be attributed to increased oxidative stress and alterations in redox signaling due to selenoprotein impairment. To date, little information is available on the extent of genetic variability in selenoproteins within populations and the possible impact of particular genetic variants of selenoproteins on cancer risk. In the present study, we sought to identify common genetic variants among six selenoproteins in the Polish population and to evaluate whether or not these variants were associated with the risk of lung and laryngeal cancer. We asked also to what extent variation in the serum level of selenium contributed to the risks of lung and laryngeal cancer in Poland.

## Materials and Methods

### Study Participants

Lung cancer cases consisted of 95 consecutive patients with lung tumors resected at our center between August 2009 and January 2011. Laryngeal cancer patients consisted of 113 consecutive patients treated for laryngeal cancer diagnosed at our center between July 2009 and January 2011. Patients were not eligible to be cases if they were diagnosed with any malignancy in the past. Patients who were treated for cancer prior to blood sampling were also ineligible.

Controls were part of a population-based study of the 1.3 million inhabitants of West Pomerania designed to identify familial aggregations of cancer performed by our center. For selenium level estimation for each case, one control was selected. Controls were matched to cases with respect to year of birth (+/−3 years), sex, total number of lung or laryngeal cancers among first degree relatives, total number of cancers among first-degree relatives, and smoking (pack-years). It was possible to find a suitable control for 86 of the 95 lung cancer cases and for 87 of the 113 laryngeal cancer cases. For estimation of genetic polymorphisms in selenoprotein genes each of the 95 lung cancer and 113 laryngeal cancer case was matched to 2 healthy controls. Matching included year of birth (+/−3 years), sex and smoking (pack-years). Cancer-free control individuals meeting matching criteria were identified by a review of the records of the population based study and invited for an interview and blood donation.

The study was approved by the Ethics Committee of the Pomeranian Medical University in Szczecin, Poland and all participants gave informed written consent.

### Measurement of Selenium Level

Selenium concentration in blood plasma was determined in 86 lung cancer and 87 laryngeal cancer pairs using graphite furnace atomic absorption spectrometry (GFAAS). Measures were validated using reference material (lyophilized human reference serum samples of Serenoform™ from Nycomed Pharma AS, Oslo, Norway). For every four samples, a reference sample was measured to keep a constant control on the quality of the measurements. Cases and controls were tested alternately. The mean drift was used as a correction value for the samples, whereas a measured drift larger than 5% from the reference material caused the device to be re-calibrated and the previous samples to be re-measured. For 30 randomly selected individuals, the selenium measurement was performed in both plasma and serum. No differences in selenium level were observed depending on type of biological material studied.

### Sequencing of Selenoprotein Genes

As the initial step, we sought to identify common genetic variants in six selenoprotein genes: *GPX1*, *GXP2*, *GXP3*, *GPX4*, *TXNRD1* and *TXNRD2*. All exons and the flanking regions were sequenced in 50 unrelated Polish individuals. We identified 35 distinct sequence variants in the six genes. Three of these had a minor allele frequency in excess of 20% and were selected for further study (rs1050450 in *GPX1*, rs713041 in *GPX4* and rs1139793 in *TXNRD2*). One other variant (rs5845 in *SEP15*) has been reported previously in association with cancer [13], [14] and was also selected for further study. We then designed Realtime-PCR assays for each of the four candidate variants. The Realtime-PCR reaction was carried out with an LightCycler® 480 (Real-Time PCR System Roche diagnostics). Each variant defined one heterozygote and two homozygote genotypes. We then determined the genotypes for all 208 cases and 416 matched controls using these assays.

### Statistics

The mean levels of selenium were compared for cases and controls using Student t-test. The associations between different categorical levels of selenium and disease status were evaluated by estimating odds ratios (OR) with 95% confidence intervals (CI) using conditional logistic regression for matched pairs. For these estimates, individuals were assigned to one of four categories, based on the distribution of selenium levels in tested groups, roughly corresponding to quartiles. The odds ratio was constructed with regard to the reference category, which was the lowest level. Each of the four tested variants was associated with three genotypes. The odds ratios for the two less frequent genotypes were generated using the most common genotype as the reference group. Samples with missing genotype in any of the four tested variants were excluded from analysis.

## Results

For the subjects in both the lung cancer and laryngeal cancer sub-studies, the cases and controls were well-matched for age, sex and smoking histories (Tables 1 and 2). Among controls (both subgroups combined) the mean serum selenium level was 75.2 µg/l for men and was 76.8 µg/l for women. The mean serum selenium level was 73.3 µg/l among current smokers, was 75.4 µg/l among past smokers and was 81.4 µg/l among never smokers. Among cases, the mean serum selenium level was 61.7 µg/l among current smokers, was 64.1 µg/l among past smokers and was 62.9 µg/l among never smokers.

**Table 1:** Characteristics of Cases and Controls for Lung Cancer Study.

Characteristic	Cases (n = 86)	Controls (n = 86)	p-value
Mean year of birth (range)	1948.3 (1931–65)	1948.3 (1933–63)	0.95
Mean age at sample (range)	61.6 (46–78)	61.9 (47–77)	0.77
Sex	n (%)		
M (%)	41 (47.7%)	41 (47.7%)	
F (%)	45 (52.3%)	45 (52.3%)	
Smoking (past/current)			
No (%)	10 (11.6%)/60 (69.8%)	13* (15.1%)/60 (69.8%)	
Yes (%)	76* (88.4%)/26 (30.2%)	73 (84.9%)/26 (30.2%)	
Pack-years (range)	22.1 (0–80)	21.5 (0–70)	0.88
Selenium concentration in µg/l (range)	63.2 (29.0–105.6)	74.6 (32.4–115.7)	<0.0001

* three never smoking controls were matched to lung cancer patients smoking <1pack-year.

**Table 2:** Characteristics of Cases and Controls for Laryngeal Cancer Study.

Characteristic	Cases (n = 87)	Controls (n = 87)	p-value
Mean year of birth (range)	1949.9 (1929–69)	1950.4 (1931–68)	0.64
Mean age at sample (range)	59.9 (41–81)	59.9 (42–79)	0.97
Sex			
M (%)	73 (83.9%)	74 (85.1%)	
F (%)	14 (16.1%)	13 (14.9%)	
Smoking (past/current)			
No (%)	1 (1.2%)/43 (49.4%)	1 (1.2%)/39 (44.8%)	
Yes (%)	86 (98.8%)/44 (50.6%)	86 (98.8%)/48 (55.2%)	
Pack-years (range)	33.0 (0–60)	30.8 (0–75)	0.24
Selenium concentration in µg/l (range)	64.8 (33.6–114.6)	77.1 (44.6–129.3)	<0.0001

Among lung cancer cases, the mean selenium level was 63.2 µg/l compared to a mean level of 74.6 µg/l for their matched controls (p<0.0001). Compared to a serum selenium value in the lowest category (≤60 µg/l) a selenium level in the highest category (>80 µg/l) was associated with a risk reduction of 90% (OR 0.10; 95% CI 0.03 to 0.34; p = 0.0002) for lung cancer (Table 3).

**Table 3:** Serum selenium levels and the risk of lung cancer.

Selenium level	Cases (%) n = 86	Controls (%) n = 86	OR (95% CI)	p-value
≤60	37 (40.0)	12 (14.0)	1	
61–70	23 (26.7)	18 (20.9)	0.40 (0.15–1.09)	0.07
71–80	12 (14.0)	29 (33.7)	0.11 (0.03–0.33)	0.0001
>80	14 (16.3)	27 (31.4)	0.10 (0.03–0.34)	0.0002

Among laryngeal cancer cases, the mean selenium level was 64.8 µg/l, compared to a mean level of 77.1 µg/l for their matched controls (p<0.0001). Compared to a serum selenium value in the lowest category (≤60 µg/l) a selenium level in the highest category (>80 µg/l) was associated with a risk reduction of 77% (OR 0.23; 95% CI 0.09 to 0.56; p = 0.001) for laryngeal cancer (Table 4).

**Table 4:** Serum selenium levels and the risk of laryngeal cancer.

Selenium level	Cases (%) n = 87	Controls (%) n = 87	OR (95% CI)	p-value
≤60	33 (37.9)	13 (14.9)	1	
61–70	23 (26.4)	16 (18.4)	0.59 (0.21–1.65)	0.32
71–80	15 (17.2)	23 (26.4)	0.35 (0.14–0.87)	0.02
>80	16 (18.4)	35 (40.3)	0.23 (0.09–0.56)	0.001

In four selenoproteins studied here we found a modest associations of rs1050450 in *GPX1* with lung and laryngeal cancer risk. (Tables 5 and 6).

**Table 5:** Genotypes for selected selenoproteins and the risk of lung cancer.

	Cases (%) n = 95	Controls (%) n = 176	OR (95% CI)	p-value
*GPX1* (rs1050450)				
CC	53 (55.8)	79 (44.9)	1	
CT	33 (34.7)	83 (47.2)	0.54 (0.31–0.95)	0.03
TT	9 (9.5)	14 (7.9)	0.85 (0.33–2.19)	0.73
*GPX4* (rs713041)				
CC	39 (41.1)	54 (30.7)	1	
CT	47 (49.5)	97 (55.2)	0.71 (0.41–1.22)	0.21
TT	9 (9.5)	25 (14.2)	0.47 (0.19–1.17)	0.11
*SEP15* (rs5845)				
GG	54 (56.8)	100 (56.8)	1	
AG	35 (36.8)	68 (38.6)	1.03 (0.62–1.72)	0.91
AA	6 (6.3)	8 (4.5)	1.52 (0.51–4.5)	0.45
*TXNRD2* (rs1139793)				
GG	55 (57.9)	109 (61.9)	1	
AG	33 (34.7)	56 (31.8)	1.16 (0.69–1.95)	0.58
AA	7 (7.4)	11 (6.3)	1.27 (0.49–3.32)	0.63

**Table 6:** Genotypes for selected selenoproteins and the risk of laryngeal cancer.

	Cases (%) n = 111	Controls (%) n = 213	OR (95% CI)	p-value
*GPX1* (rs1050450)				
CC	65 (58.6)	97 (45.5)	1	
CT	37 (33.4)	95 (44.6)	0.51 (0.29–0.9)	0.02
TT	9 (8.1)	21 (9.8)	0.83 (0.28–2.41)	0.73
*GPX4* (rs713041)				
CC	42 (37.8)	68 (31.9)	1	
CT	46 (41.5)	108 (50.7)	0.9 (0.5–1.61)	0.72
TT	23 (20.7)	37 (17.4)	1.15 (0.53–2.48)	0.73
*SEP15* (rs5845)				
GG	61 (54.9)	128 (60.0)	1	
AG	47 (42.4)	73 (34.3)	1.51 (0.85–2.66)	0.16
AA	3 (2.7)	12 (5.6)	0.4 (0.08–2.06)	0.27
*TXNRD2* (rs1139793)				
GG	57 (51.4)	117 (54.9)	1	
AG	44 (39.6)	82 (38.5)	1.26 (0.73–2.18)	0.41
AA	10 (9.0)	14 (6.6)	1.72 (0.55–5.34)	0.35

## Discussion

In our study, we sought to see if systemic differences in selenium levels occur in individuals with lung and laryngeal cancer and healthy controls in northwestern Poland. For both cancer sites, the cancer risk decreased with increasing levels of circulating selenium and the dose-response curve appeared linear. There was no evidence that there was a threshold above which the association with serum selenium was disrupted or reversed. The mean selenium level in the Polish control population was 75.8 µg/l. Only 21% (18/86) of the lung cancer cases and 28% (24/87) of the laryngeal cancer cases exhibited a level at or above this control mean. Compared to individuals in the category from 70 µg/l to 80 µg/l, those with a level below 60 µg/l were at roughly ten-fold increased risk for lung cancer and a three-fold increased risk of laryngeal cancer.

The mean level of selenium in the controls was less than optimal according the proposed level of 120 µg/l to be optimal for proper selenoprotein expression and activity [1]. In our study, only one control (and none of cases) had a level that exceeded 120 µg/l.

Several case-control and cohort studies have been conducted up to date to assess the influence of selenium on lung cancer risk. Some of these studies suggested that selenium may have a protective effect against lung cancer [15]–[18]. However, other studies have failed to confirm this observation, either showing no association [19]–[23] or an adverse effect [24], [25] of higher selenium level on incidence of lung cancer. These studies differed in study design (population or nested-case-control, with or without selenium supplementation), low or high baseline population selenium level, methods of selenium measurements (dietary intake, toenail or serum level) and the length of the follow-up period. A meta-analysis of 16 studies on selenium and lung cancer showed the pooled RR 0.74 (95% CI 0.57–0.97) for subjects with higher selenium exposures. The protective effect was greater for studies from areas with lower average baseline selenium level (RR 0.72) than from areas with higher average selenium level (RR 0.86) [26]. The possibility that the protective effect of selenium may be limited to individuals with a low baseline level is supported by the NPC trial [8], [9]. In the NPC study, American subjects were randomized to receive either selenium 200 µg/day versus placebo [8]. After 7.9 years of follow-up, the odds ratio for incident lung cancer was 0.70 (95% 0.40 to 1.21; p = 0.18). Notably, the benefit was limited to individuals with low baseline plasma selenium (<106 µg/l). In this subgroup the association with selenium supplementation was 0.42 (95% CI 0.18 to 0.96). Of interest, at this cutoff point, 96% (166/173) of Polish controls would fall into this category. In the recently published SELECT trial [27], there was no benefit for men randomized to selenium 200 µg/l versus placebo in terms of incident lung cancers (HR 1.12; 95% CI 0.73 to 1.72). However, in that study, the mean baseline selenium level was 137 µg/l and data were not subdivided by serum selenium.

There are several limitations to our study. Perhaps the most important limitation of our case-control study is that the selenium measurements for cases were made after the diagnosis of lung or laryngeal cancer, but before therapy. It has been reported that acute and chronic injury and infections alter concentration of variety of micronutrients [28], [29]. As in this study the selenium concentration in cancer patients was measured after they were diagnosed, it is possible that the detected low selenium level could be a consequence of their disease. If this were the cases, then a low selenium level would be a marker of risk rather than a risk factor.

The sample size in our study is relatively small but the associations were strong and highly significant. The cases and controls were matched on smoking status and it is therefore not likely that the association could be confounded by smoking, but it is possible that another confounding variable could introduce bias.

In four selenoproteins studied here we found some evidence of modest association of genetic variant in *GPX1* with lung and laryngeal cancer risk. The influence of rs1050450 in *GPX1* on lung cancer risk have been already analyzed in several studies, but results were inconsistent [30]–[34]. In the current study we found an indication of association of T-allele with reduction of lung and laryngeal cancer risk, however these results were of borderline significance.

Our study indicates that a low selenium level is significantly associated with up to a tenfold higher risk of lung and laryngeal cancer for individuals in the lowest category of selenium. Ideally this association would be confirmed in a large prospective study. If confirmed, it is possible that low serum selenium level will appear to be a strong indicator for the risk of lung and laryngeal cancer, then this will be an important potential target of therapeutic intervention.

## References

[1] Rayman MP (2005) Selenium in cancer prevention: a review of the evidence and mechanism of action. Proc of the Nutrition Society 64: 527–542. 10.1079/pns2005467 16313696

[2] Rayman MP (2012) Selenium and human health. Lancet 379: 1256–1268. 22381456 10.1016/S0140-6736(11)61452-9

[3] Nomura A, Heilbrun LK, Morris JS, Stemmermann GN (1987) Serum selenium and the risk of cancer, by specific sites: case–control analysis of prospective data. J Natl Cancer Inst 79: 103–108. 3474437

[4] Navarro Silvera SA, Rohan TE (2007) Trace elements and cancer risk: a review of the epidemiologic evidence. Cancer Causes Control 18: 7–27. 17186419 10.1007/s10552-006-0057-z

[5] Ratnasinghe D, Tangrea JA, Forman MR, Hartman T, Gunter EW, et al (2000) Serum tocopherols, selenium and lung cancer risk among tin miners in China. Cancer Causes Control 11: 129–135. 10710196 10.1023/a:1008977320811

[6] Hartman TJ, Taylor PR, Alfthan G, Fagerstrom R, Virtamo J, et al (2002) Toenail selenium concentration and lung cancer in male smokers. Cancer Causes Control 13: 923–928. 12588088 10.1023/a:1021912117067

[7] van den Brandt PA, van’t Veer P, Goldbohm RA, Dorant E, Volovics A, et al (1993) A prospective study on selenium status and the risk of lung cancer. Cancer Res 53: 4860–4865. 8402674

[8] Reid ME, Duffield-Lillico AJ, Garland L, Turnbull BW, Clark LC, et al (2002) Selenium supplementation and lung cancer incidence: an update of the nutritional prevention of cancer trial. Cancer Epidemiol Biomark Prev 11: 1285–1291. 12433704

[9] Duffield-Lillico AJ, Reid ME, Turnbull BW, Combs GF Jr, Slate EH, et al (2002) Baseline characteristics and the effect of selenium supplementation on cancer incidence in a randomized clinical trial: a summary report of the Nutritional Prevention of Cancer Trial. Cancer Epidemiol Biomarkers Prev 11: 630–639. 12101110

[10] Zhou L, Huang Y, Wang Z, Ye L, Hou W, et al (2011) Serum and lung tissue selenium measurements in subjects with lung cancer from Xuanwei, China. Zhongguo Fei Ai Za Zhi 14: 39–42. 21219830 10.3779/j.issn.1009-3419.2011.01.08 PMC5999700

[11] Kryukov GV, Castellano S, Novoselov SV, Lobanov AV, Zehtab O, et al (2003) Characterization of mammalian selenoproteomes. Science 300: 1439–1443. 12775843 10.1126/science.1083516

[12] Reeves MA, Hoffmann PR (2009) The human selenoproteome: recent insights into functions and regulation. Cel Mol Life Sci 66: 2457–2478. 10.1007/s00018-009-0032-4 PMC2866081 19399585

[13] Sutherland A, Kim DH, Relton C, Ahn YO, Hesketh J (2010) Polymorphisms in the selenoprotein S and 15-kDa selenoprotein genes are associated with altered susceptibility to colorectal cancer. Genes Nutr 5: 215–223. 21052528 10.1007/s12263-010-0176-8 PMC2935533

[14] Jablonska E, Gromadzinska J, Sobala W, Reszka E, Wasowicz W (2008) Lung cancer risk associated with selenium status is modified in smoking individuals by Sep15 polymorphism. Eur J Nutr 47: 47–54. 18239845 10.1007/s00394-008-0696-9

[15] Knekt P, Aromaa A, Maatela J, Alfthan G, Aaran RK, et al (1990) Serum selenium and subsequent risk of cancer among Finnish men and women. J Natl Cancer Inst 82: 864–868. 2332904 10.1093/jnci/82.10.864

[16] Kabuto M, Imai H, Yonezawa C, Neriishi K, Akiba S, et al (1994) Prediagnostic serum selenium and zinc levels and subsequent risk of lung and stomach cancer in Japan. Cancer Epidemiol Biomarkers Prev 3: 465–469. 8000296

[17] Knekt P, Marniemi J, Teppo L, Heliövaara M, Aromaa A (1998) Is low selenium status a risk factor for lung cancer? Am J Epidemiol 148: 975–982. 9829869 10.1093/oxfordjournals.aje.a009574

[18] Comstock GW, Alberg AJ, Huang HY, Wu K, Burke AE, et al (1997) The risk of developing lung cancer associated with antioxidants in the blood: ascorbic acid, carotenoids, alpha-tocopherol, selenium, and total peroxyl radical absorbing capacity. Cancer Epidemiol Biomarkers Prev 6: 907–916. 9367064

[19] Menkes MS, Comstock GW, Vuilleumier JP, Helsing KJ, Rider AA, et al (1986) Serum beta-carotene, vitamins A and E, selenium, and the risk of lung cancer. N Engl J Med 315: 1250–1254. 3773937 10.1056/NEJM198611133152003

[20] Nomura A, Heilbrun LK, Morris JS, Stemmermann GN (1987) Serum selenium and the risk of cancer, by specific sites: case-control analysis of prospective data. J Natl Cancer Inst 79: 103–108. 3474437

[21] Kromhout D. Essential micronutrients in relation to carcinogenesis. Am J Clin Nutr. 1987 May;45(5 Suppl): 1361–1367. 10.1093/ajcn/45.5.1361 3578124

[22] Goodman GE, Schaffer S, Bankson DD, Hughes MP, Omenn GS (2001) Carotene and Retinol Efficacy Trial Co-Investigators. Predictors of serum selenium in cigarette smokers and the lack of association with lung and prostate cancer risk. Cancer Epidemiol Biomarkers Prev 10: 1069–1076. 11588133

[23] Epplein M, Franke AA, Cooney RV, Morris JS, Wilkens LR, et al (2009) Association of plasma micronutrient levels and urinary isoprostane with risk of lung cancer: the multiethnic cohort study. Cancer Epidemiol Biomarkers Prev 18: 1962–1970. 19531680 10.1158/1055-9965.EPI-09-0003 PMC2755047

[24] Garland M, Morris JS, Stampfer MJ, Colditz GA, Spate VL, et al (1995) Prospective study of toenail selenium levels and cancer among women. J Natl Cancer Inst 87: 497–505. 7707436 10.1093/jnci/87.7.497

[25] Ratnasinghe D, Tangrea JA, Forman MR, Hartman T, Gunter EW, et al (2000) Serum tocopherols, selenium and lung cancer risk among tin miners in China. Cancer Causes Control 11: 129–135. 10710196 10.1023/a:1008977320811

[26] Zhuo H, Smith AH, Steinmaus C (2004) Selenium and lung cancer: a quantitative analysis of heterogeneity in the current epidemiological literature. Cancer Epidemiol Biomarkers Prev 13: 771–778. 15159309

[27] Lippman SM, Klein EA, Goodman PJ, Lucia MS, Thompson IM, et al (2009) Effect of selenium and vitamin E on risk of prostate cancer and other cancers: the Selenium and Vitamin E Cancer Prevention Trial (SELECT). JAMA 301: 39–51. 19066370 10.1001/jama.2008.864 PMC3682779

[28] Renko K, Hofmann PJ, Stoedter M, Hollenbach B, Behrends T, et al (2009) Down-regulation of the hepatic selenoprotein biosynthesis machinery impairs selenium metabolism during the acute phase response in mice. FASEB J 23: 1758–1765. 19136613 10.1096/fj.08-119370

[29] Duncan A, Talwar D, McMillan DC, Stefanowicz F, O’Reilly DS (2012) Quantitative data on the magnitude of the systemic inflammatory response and its effect on micronutrient status based on plasma measurements. Am J Clin Nutr 95: 64–71. 22158726 10.3945/ajcn.111.023812

[30] Ratnasinghe D, Tangrea JA, Andersen MR, Barrett MJ, Virtamo J, et al (2000) Glutathione peroxidase codon 198 polymorphism variant increases lung cancer risk. Cancer Res 60: 6381–6383. 11103801

[31] Yang P, Bamlet WR, Ebbert JO, Taylor WR, de Andrade M (2004) Glutathione pathway genes and lung cancer risk in young and old populations. Carcinogenesis 25: 1935–1944. 15192016 10.1093/carcin/bgh203

[32] Skuladottir H, Autrup H, Autrup J, Tjoenneland A, Overvad K, et al (2005) Polymorphisms in genes involved in xenobiotic metabolism and lung cancer risk under the age of 60 years. A pooled study of lung cancer patients in Denmark and Norway. Lung Cancer. 48: 187–199. 10.1016/j.lungcan.2004.10.013 15829318

[33] Raaschou-Nielsen O, Sørensen M, Hansen RD, Frederiksen K, Tjønneland A, et al (2007) GPX1 Pro198Leu polymorphism, interactions with smoking and alcohol consumption, and risk for lung cancer. Cancer Lett 247: 293–300. 16797832 10.1016/j.canlet.2006.05.006

[34] Rosenberger A, Illig T, Korb K, Klopp N, Zietemann V, et al (2008) Do genetic factors protect for early onset lung cancer? A case control study before the age of 50 years. BMC Cancer 8: 60. 18298806 10.1186/1471-2407-8-60 PMC2292731

