# Correction: A Low Selenium Level Is Associated with Lung and Laryngeal Cancers

**DOI:** 10.1371/annotation/f777aaec-b6b8-4480-9cce-18e0f1b8e5d5

**Published:** 2013-08-16

**Authors:** Katrzyna Jaworska, Satish Gupta, Katarzyna Durda, Magdalena Muszyńska, Grzegorz Sukiennicki, Ewa Jaworowska, Tomasz Grodzki, Mieczysław Sulikowski, Piotr Waloszczyk, Janusz Wójcik, Jakub Lubiński, Cezary Cybulski, Tadeusz Dębniak, Marcin Lener, Antoni W. Morawski, Karol Krzystolik, Steven A. Narod, Ping Sun, Jan Lubiński, Anna Jakubowska

The correct name of the ninth author is: Piotr Waloszczyk.

The correct affiliation for this author is: Independent Laboratory of Pathology, Zdunomed, Szczecin, Poland. 

